# Review of Image Quality Assessment Methods for Compressed Images

**DOI:** 10.3390/jimaging10050113

**Published:** 2024-05-08

**Authors:** Sonain Jamil

**Affiliations:** Department of Computer Science, Norwegian University of Science and Technology (NTNU), 2815 Gjovik, Norway; sonainjamil@ieee.org or sonainj@stud.ntnu.no

**Keywords:** image quality assessment, image quality metrics, JPEG AIC, compression artifacts, LLMs

## Abstract

The compression of images for efficient storage and transmission is crucial in handling large data volumes. Lossy image compression reduces storage needs but introduces perceptible distortions affected by content, compression levels, and display environments. Each compression method generates specific visual anomalies like blocking, blurring, or color shifts. Standardizing efficient lossy compression necessitates evaluating perceptual quality. Objective measurements offer speed and cost efficiency, while subjective assessments, despite their cost and time implications, remain the gold standard. This paper delves into essential research queries to achieve visually lossless images. The paper describes the influence of compression on image quality, appropriate objective image quality metrics (IQMs), and the effectiveness of subjective assessment methods. It also provides an overview of the existing literature, surveys, and subjective and objective image quality assessment (IQA) methods. Our aim is to offer insights, identify challenges in existing methodologies, and assist researchers in selecting the most effective assessment approach for their needs.

## 1. Introduction

Image compression is essential for efficiently storing and distributing massive amounts of data, including photographs and videos. While producing distortions in picture data that may be perceptible to the human eye based on the content and degree of compression, lossy image compression lowers storage needs [1]. All compression methods result in different visual artifacts, including color shift, blocking, blurring, or ringing irregularities, among others.

It is essential to undertake perceptual quality evaluation studies to determine the severity of the added visual artifacts to standardize a new and efficient lossy compression approach [2,3]. Nowadays, it is normal to share and gather a lot of photos every day. Consequently, the necessity for creative picture compression methods to reduce storage space is constant. A sound approach to assessing the effectiveness of compression methods is crucial in this situation. Such performance is often evaluated using objective quality measurements, which are quick and affordable but not necessarily accurate [4]. However, subjective image quality evaluation experiments, which are costly and time-consuming but trustworthy because they rely on the subjective judgment of many subjects, are the most effective means of assessing how well image compression techniques work [5]. Notwithstanding their advantages and disadvantages, both techniques are equally important for the IQA of the pictures that have been compressed using various compression standards, including JPEG 1 [6], JPEG 2000 [7], JPEG XL [8], JPEG AI [9], etc. This leads to several research questions to find visually lossless images.

- What is the impact of compression on image quality? This question explores how various compression algorithms influence key visual elements and how these changes affect user perception and application usability.
- Which objective IQMs are suitable for finding visually lossless compressed images? We investigate the metrics that best correlate with human perception, highlighting their effectiveness in different scenarios.
- Which subjective method is the most efficient and robust? This inquiry assesses the practicality of subjective methods, comparing their reliability and resource demands in diverse usage contexts.

To answer these questions, we present a review of the impact of the different JPEG image compression standards on image quality in this paper. We also summarize the existing reviews and surveys on the image quality assessment. We analyze existing subjective IQA methods as well as objective IQMs. We also present knowledge gained and our insights from the analysis. We highlight open research challenges in the existing methods. This study will assist researchers in conducting subjective experiments to assess the quality of compressed images and choose the best approach for their use case more effectively.

**Figure 1:**
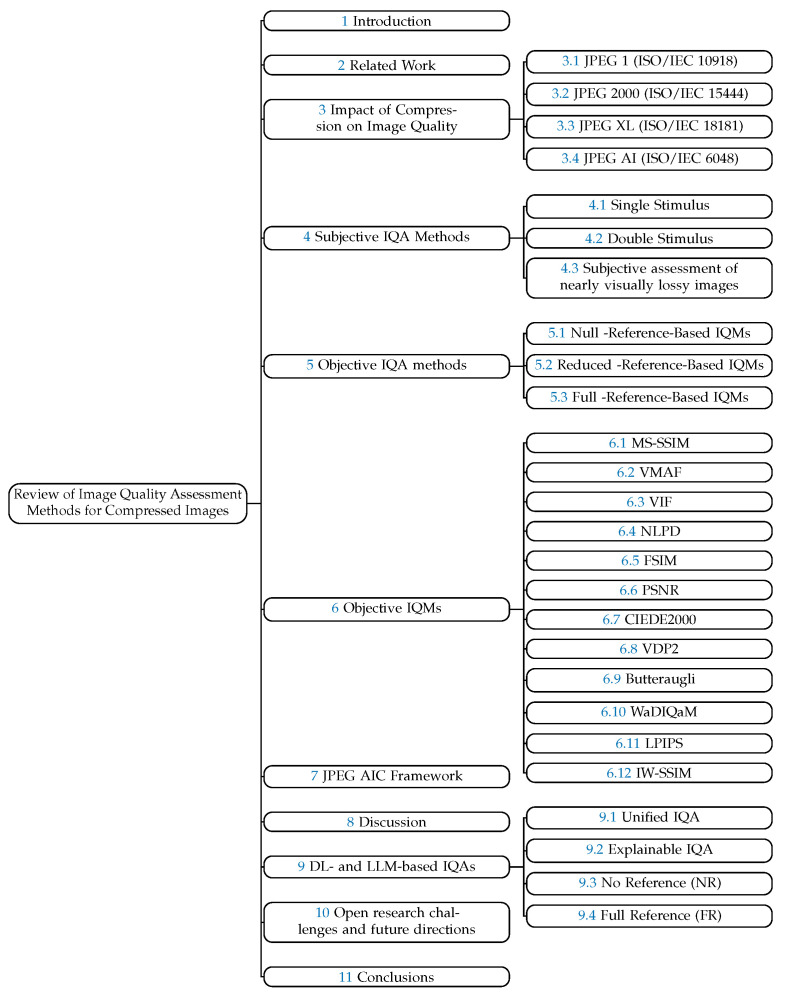
Organization of the survey.

Figure 1 presents the organization of the paper. The rest of the paper is organized as follows: Section 2 summarizes the existing literature reviews and surveys on image quality assessment methods. Section 3 explains the impact of different image compression standards, i.e., JPEG, JPEG 2000, JPEG XL, and JPEG AI, on perceptual image quality. Section 4 focuses on the subjective IQA methods, whereas Section 5 focuses on the objective IQA methods. Similarly, Section 6 describes objective IQMs. This section is followed by Section 7, which focuses on the JPEG AIC framework. Then, Section 8 presents the discussion about the key takeaways. After that, Section 9 presents IQA methods based on deep learning (DL) and large language models (LLMs). After that, Section 10 provides insight into open research challenges and future directions. Finally, Section 11 concludes the work.

## 2. Related Work

Numerous survey articles have delved into the realm of image quality assessment (IQA) methods, offering valuable insights into both subjective and objective evaluation methodologies. One such recent endeavor, undertaken by Leveque et al. [10], conducted a comparative study specifically within the medical images domain, scrutinizing subjective methodologies. Similarly, Opozda and Sochan conducted a comprehensive examination of both subjective and objective methods for 2D and 3D image types [11]. Expanding this domain, Ouni et al. explored existing subjective and objective evaluation methods for images and videos [12]. Other studies, such as Lee et al. [13], have ventured into comparing subjective quality evaluation approaches, even in controlled laboratory settings, while Pinson et al. [14] explored six different subjective video quality rating approaches. This study’s distinctive contribution lies in its extensive review of subjective and objective quality evaluation approaches and standards, particularly focusing on the quality assurance of compressed images. Furthermore, the paper delves into the assessment of subjective methods for identifying nearly visually lossless or just noticeable artifacts in compressed images. It does not stop there, as it also addresses the impact of various testing environments, such as controlled rooms, laboratories, or crowdsourced setups, on subjective testing outcomes. Ultimately, the paper concludes with recommendations for effectively conducting both subjective and objective evaluations of image quality tasks.

While several surveys in the field of image quality evaluation have honed in on either subjective or objective methodologies, these studies often pertain to different domains like medical images, image restoration, or image inpainting. Howev er, there remains a gap in the literature: a comprehensive exploration of both the subjective and objective aspects of image analysis approaches.

Lin et al. [15], for example, conducted a comprehensive evaluation of perceptual visual quality factors with the goal of predicting picture quality based on human perception. Through their work, several computational modules were introduced, such as frequent feature and artifact detection, visual attention, barely noticeable distortion, and signal breakdown.

In a similar vein, Mantiuk1 et al. [16] examined crucial steps in image analysis, including posterior power analysis, statistical testing, and confidence interval establishment. They examined two approaches to rating findings, with a focus on information of practical and statistical value. The forced-choice pairwise comparison approach had the least measurement variation and, thus, the best accurate results among the four major subjective quality evaluation methods they studied.

Furthermore, Lui et al. [17] investigated the use of visual quality grading in perceptual coding, evaluating the performance of cutting-edge visual quality metrics through benchmarking.

Looking ahead, the future of visual quality rating is contemplated. Kamble et al. [18] presented an overview of existing approaches for evaluating no-reference image quality. Their study encompassed various aspects such as the types of noise and distortions addressed, the algorithmic techniques and parameters employed, the databases used for algorithm assessment, and benchmarking against both other algorithms and the human visual system.

Chow et al. [19] contributed to this landscape by offering an overview of methods for evaluating no-reference image quality. They considered factors like the types of noise and distortions addressed, the approaches and settings used by these algorithms, the databases utilized for their evaluation, and their benchmarked performance relative to other algorithms and human visual perception.

The summary of the surveys and review articles discussed in this section is presented in Table 1.

Based on the selection criteria outlined in Algorithm 1, we selected papers for analysis.
**Algorithm 1** Article Selection Criteria**Require:** Search databases **Ensure:** Articles from 2017 to 2024   **while** keyword: image quality assessment (IQA) for compressed images or JPEG AIC or image quality assessment methods **do**       **if** Discusses IQA methods|Evaluate IQA|Analyze IQA based on LLMs **then**            Consider for analysis       **else if** Does not discuss IQA for compressed images **then**            Exclude from the analysis       **end if**   **end while**

## 3. Impact of Compression on Image Quality

This section briefly discusses the types of distortions that are being introduced by the different JPEG standards.

### 3.1. JPEG 1 (ISO/IEC 10918)

JPEG 1 is a DCT-based image compression standard; all DCT-based compression techniques result in “block artifacts” or visible distortions in the compressed image that resemble squares. These artifacts arise from the division of the image into blocks for the DCT process; when the image is compressed, the boundaries between these blocks become visible, degrading the overall visual quality of the image [6].

### 3.2. JPEG 2000 (ISO/IEC 15444)

JPEG 2000 uses DWT instead of DCT, as opposed to JPEG 1, in order to provide superior compression efficiency and handling of high-resolution images. DWT-based compression techniques, such as JPEG 2000, however, frequently result in blur problems. These artifacts degrade the image’s overall quality by causing a loss of sharpness or detail. JPEG 2000 also has particular problems with ringing and halo effects, as well as tiling artifacts. When images are processed, they are divided into smaller parts or tiles, which might result in obvious seams or discontinuities. This is when tiling artifacts arise. Additional distortions that affect an image’s visual fidelity are ringing and halo effects, which show up as strange, oscillating patterns or bright outlines surrounding things. Even though these abnormalities differ from those in the original JPEG standard, they nevertheless jeopardize the compressed images’ integrity and beauty [7].

### 3.3. JPEG XL (ISO/IEC 18181)

JPEG XL is a more recent and sophisticated image compression standard that expands upon the capabilities and flexibility of JPEG 1. It uses a DCT-based variable and modular method, which makes the compression process more flexible and effective. The fact that JPEG XL supports both lossy and lossless compression modes is one of its primary characteristics. As observed in the original JPEG 1 standard, block artifacts are a prevalent problem with DCT-based techniques, and JPEG XL can still create them in its lossy form. In the photograph, these artifacts can be seen as obvious distortions that resemble blocks. However, a notable improvement in JPEG XL is that the intensity of these block artifacts is significantly reduced compared to JPEG 1, resulting in better image quality.

Apart from causing block distortions, JPEG XL compression may result in additional kinds of image deterioration. These include color bleeding (where colors appear to leak or extend beyond their bounds), ringing (the appearance of spurious or oscillating patterns around edges), and softening (a small blurring or loss of sharpness). Though the degree to which each of these artifacts degrades the image’s overall visual quality varies according to the compression setting and type of image being compressed, all of them have an impact. The improvements in JPEG XL are designed to strike a compromise between compression effectiveness and image quality, offering a contemporary approach that overcomes some of the drawbacks of previous JPEG standards [8].

### 3.4. JPEG AI (ISO/IEC 6048)

JPEG AI is the most recent advancement in image compression technology, using a cutting-edge, learning-based methodology. In contrast to conventional JPEG standards, which rely on mathematical formulas such as DCT or DWT, JPEG AI makes use of a deep learning model called a convolutional auto-encoder. Convolutional auto-encoders are made to learn patterns and features straight from the input, which allows them to compress and subsequently decompress (reconstruct) images effectively.

The auto-encoder in this sophisticated model initially reduces the image’s size considerably by compressing it into a compact representation. This procedure works especially well at low bit rates, which is important for applications with constrained bandwidth or storage. But utilizing this method has a significant trade-off: the reconstructed image will have a particular kind of distortion called “striped region distortion”, especially at these lower bit rates [20]. The visual quality and integrity of a picture can be impacted by striped region distortion, which appears as bands or stripes in patterns. This artifact, which arises from the way the convolutional auto-encoder interprets and reconstructs the picture input, illustrates the difficulties of learning-based approaches in striking a balance between image integrity and compression efficiency. In spite of this, JPEG AI represents a major advancement in picture compression by utilizing machine learning to attain high degrees of effectiveness and versatility [9].

***Key Takeaways:** From the above discussion, it is evident that the most common type of artifact introduced by JPEG 1 is a blocking artifact, whereas the most common type of artifact introduced by JPEG 2000 is a ringing artifact. Similarly, JPEG XL mostly introduces softening and ringing artifacts, and finally, the most common artifact introduced by JPEG AI is striped region distortion at an extremely low bit rate.*

## 4. Subjective IQA Methods

Subjective methods for assessing image quality are regarded as reliable approaches. These methods involve human subjects observing images on displays and expressing their opinions about image quality using various predefined scales [21]. These subjective tests adhere to several standards that ensure the credibility of image quality evaluations [22,23].

Standards for subjective tests with television pictures have been established by the International Telecommunication Union Radio Communication Sector (ITU-R) BT.500-11. Testing conditions, presentation techniques, and test result assessment are all covered by these standards [11]. Furthermore, testing parameters for the subjective evaluation of video data quality have been established by ITU-T P.910 [12].

While ITU-R BT.1129-2 has defined procedures for standard video sequences, ITU-R BT.814-1 has standardized display device contrast and brightness settings for subjective testing [23,24]. Based on the stimulus used in subjective testing, ITU-R essentially offers a variety of standards that may be generally divided into two categories: stimulus and double-stimulus approaches. In single-stimulus testing, participants see just one image; in double-stimulus tests, they see two images side by side, each with a different grading scheme.

### 4.1. Single Stimulus

**Figure 2:**
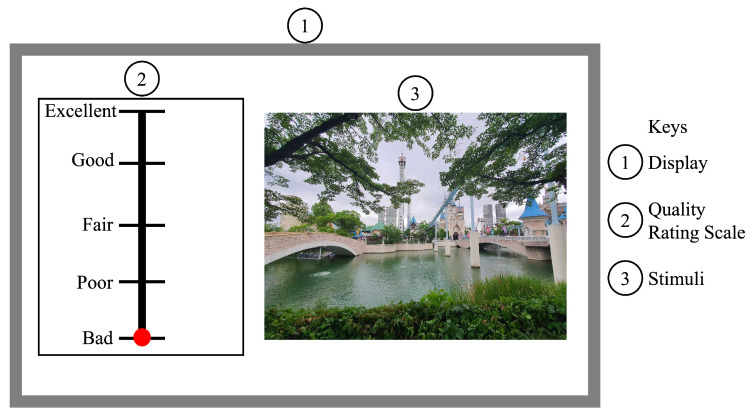
An example of the single-stimulus IQA method.

Subjects assess individual pictures in single-stimulus techniques. They go on to the next image after rating each one’s quality. Simple assessments that need minimal steps might benefit from this method. The quality of images was evaluated using single-stimulus tests by Cheng et al. [25] and Sheikh et al. [26]. Figure 2 is an example of a single-stimulus approach.

A kind of single-stimulus approach called absolute category rating (ACR) asks participants to rank the quality of images on a five-point scale: terrible, poor, fair, good, and outstanding. For a large number of photographs, this approach may be time-consuming, and the substance of the images may affect subjective judgments. Furthermore, to lessen the variance resulting from subject judgments regarding picture content, an absolute category rating with a hidden reference (ACR-HR) incorporates the original, undistorted image without the subject’s awareness. ACR-HR has been utilized in certain research studies [22,27] to assess learning-based image codecs. A different variation uses a continuous scale for grading and is called single-stimulus continuous quality assessment (SSC-QE).

### 4.2. Double Stimulus

**Figure 3:**
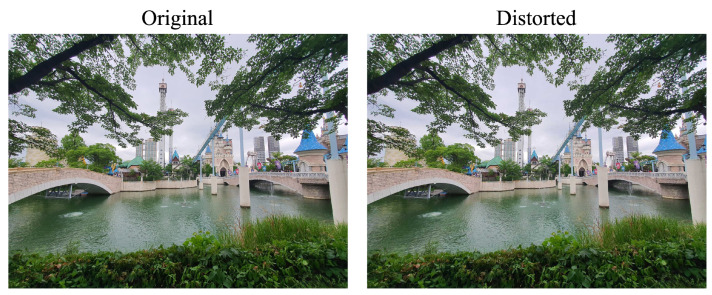
Example of double-stimulus method.

Double-stimulus techniques include presenting participants with two distinct stimuli in order to assess image distortion (Figure 3). Although these tests take longer to complete than tests with a single stimulus, they are thought to be more accurate and efficient in detecting picture distortion. Double-stimulus tests were used by Testolina et al. [28] to assess coding performance.

The double-stimulus impairment scale (DSIS) is one kind of double-stimulus technique in which participants assess picture impairment using a predetermined quality scale. Another variation is the double-stimulus continuous quality scale (DSCQS), in which participants rate both pictures’ quality on a fixed scale. Subjects evaluate the test image on a predetermined scale after comparing it to the reference image using a double-stimulus comparison scale (DSCS). When it comes to performance reviews, DSCS is thought to be the most trustworthy.

These methods are generally conducted in controlled environments with normal lighting to avoid uncertainties caused by external factors. However, crowdsource-based methods, where subjects conduct tests remotely, have gained popularity. Egger et al. [29] and Chen et al. [30] reviewed crowdsourced-based methodologies, and recent studies like Testolina et al. [28] used crowdsourcing for subjective tests on online platforms like Amazon Mechanical Turk.

***Key Takeaways:** Subjective methods are valuable for assessing image quality, involving human observers who use predefined scales to express their opinions. ITU has established standards for credible subjective evaluations, including stimulus and double-stimulus methods, addressing factors like testing environments and display settings. While single-stimulus tests are simpler but time-consuming, double-stimulus tests are more reliable for evaluating image distortion, with both types generally conducted in controlled environments but increasingly explored through crowdsource-based methods for remote testing.*

### 4.3. Subjective Assessment of Nearly Visually Lossy Images

The subjective methods discussed earlier are primarily suitable for images with noticeable visual distortions that are easily perceivable by humans. However, recent advancements in high-performance image compression techniques allow for the reconstruction of visually lossless images.

With the progress in storage devices and advanced networks, handling large amounts of data has become more manageable. This has resulted in a growing demand for effective image compression algorithms that can achieve lossless reconstruction of image data. The previously discussed subjective methods are ill-suited for standardizing these high-performing compression techniques, as they cannot detect subtle distortions or color shifts in images.

JPEG members have introduced standardized methodologies for assessing high-performance, visually lossless images to address this need. In one approach, subjects are presented with two test images alongside the original image. They are tasked with selecting the image least similar to the original at a given time. In another method, the original and reconstructed images are displayed simultaneously, with intermittent interleaving.

When noticeable distortion is present in the test image, subjects perceive flickering. Conversely, when the distortion is imperceptible, no flickering is observed. For instance, Willeme et al. [31] used the flickering test methodology to evaluate the JPEG XS standard. It is worth noting that the concept of visually lossless image compression is relatively new, and as a result, this subjective method is not as widely adopted as the previously established subjective methodologies [1].

According to JPEG AIC, three methods are currently being used for this task. These methods are the flicker test, boosted triplet comparison, and remote expert viewing.

## 5. Objective IQA Methods

These mathematical models serve the purpose of automatically estimating image quality in qualitative terms, mirroring the evaluation made by human observers. These metrics offer a practical advantage in real-time applications when compared to the costly and time-consuming subjective tests mentioned in the reference [11]. The realm of image processing and computer vision is where these metrics find versatile applications. They prove invaluable in systems designed for image quality control, allowing for the selection of image quality based on these precise metrics.

Furthermore, these metrics also play a pivotal role in evaluating image processing algorithms. By employing these metrics, it becomes possible to rank different algorithms based on their ability to produce the highest-quality images as output. This is particularly useful when making choices among multiple algorithms for specific tasks.

In addition to these applications, image quality metrics (IQMs) find applications in visual network-based image communication systems. In such systems, these IQMs are instrumental in optimizing filtering procedures at both the encoder and decoder ends, as outlined in reference [32].

The field has seen the emergence of several intelligent image quality measurement metrics, as highlighted in various evaluation studies [27]. These metrics can be categorized based on the requirement of an absolute quality reference or a distortion-free original image for quality assessment. This categorization gives rise to three main categories: null-reference-based metrics, fused full-reference-based metrics, and full-reference-based quality metrics, as elucidated in reference [24].

### 5.1. Null-Reference-Based IQMs

Null-reference-based image quality metrics possess a distinctive feature in that they do not necessitate the original referenced picture for the purpose of determining image quality [23]. To anticipate picture quality, they instead depend on calculations using image properties like brightness, contrast, and other factors. These metrics are used in a number of image communication systems, where they evaluate the quality of the picture based just on the test image, eliminating the necessity for the original image to serve as a reference.

Null-reference-based measurements are more complicated to forecast picture quality than complete-reference-based measures, though. The assessment procedure itself is further complicated by this, especially when handling missing original photos.

### 5.2. Reduced-Reference-Based IQMs

Shifting to reduced-reference-based image quality metrics, these algorithms evaluate the distorted picture quality by utilizing just a subset of the reference image’s attributes instead of the whole reference image. The test picture quality may be predicted with the use of these particular attributes. These selected characteristics embody representations of the reference pictures and are perceptually significant, which is important for assessing image quality.

### 5.3. Full-Reference-Based IQMs

Lastly, target image quality is evaluated using complete-reference-based image quality criteria by contrasting it with its original, unaltered counterpart. Measuring the distortion divergence between the reference and distorted pictures utilized in the measure yields the value for the whole reference metric.

## 6. Objective IQMs

### 6.1. Multi-Scale Structural Similarity Index (MS-SSIM)

A well-known technique for assessing picture quality is MS-SSIM. It functions by evaluating characteristics over a range of resolutions to determine the relative quality of pictures. This method produces excellent results, particularly when used with machine learning-based image codecs. MS-SSIM is more versatile than single-scale approaches like SSIM since it accounts for variations in viewing circumstances and picture resolution.

The MS-SSIM metric’s capacity to include image synthesis for adjusting the parameters that establish the relative significance of various scales in the analysis is one of its advantages. In essence, it modifies its assessment standards according to the particular features of the images that are being compared. In practical terms, a higher MS-SSIM score indicates superior image quality, making it a valuable tool for assessing and comparing images.

### 6.2. Video Multimethod Assessment Fusion (VMAF)

The quality measuring method known as VMAF was first created by Netflix. Finding artifacts resulting from compression and rescaling procedures is its main goal. VMAF uses a special way to calculate scores using many quality evaluation techniques, and then it combines these values using a support vector machine (SVM) to produce the quality score.

Even though VMAF was first developed to evaluate the quality of movies and videos, it has also shown to be a highly useful tool for analyzing individual images. It works especially well with image codecs that use machine learning methods. The input photos must, however, be in the YUV color space format for VMAF to function. FFMPEG, which follows the BT.709 primaries, can convert PNG pictures in the RGB color space into the necessary YUV 4:4:4 format.

Better picture quality is indicated by a higher VMAF score, just as in other image quality metrics. Therefore, despite the fact that its initial intent was to evaluate movies, it is an invaluable tool for evaluating and comparing the quality of photos [33].

### 6.3. Visual Information Fidelity (VIF)

The VIF metric is a technique used to measure the amount of information lost during procedures such as picture compression that is seen by humans. The main objective of VIF is to evaluate the degree to which an image’s information is accurately retained during deterioration. By examining natural scene data and creating a link using the Shannon mutual information shared by the original, perfect image and the degraded image, it does this.

The VIF metric functions in the wavelet domain, which is one of its noteworthy characteristics. As a result, it analyzes the information content of the picture across a range of frequency ranges, which can offer important insights into how deterioration affects certain image characteristics.

Numerous studies have shown that the values of the VIF measure closely correspond with how people perceive the quality of a picture. This is valid for both contemporary learning-based image codecs and conventional picture compression. VIF is a valuable technique for quantitatively evaluating and comparing the quality of pictures that have experienced different sorts of degradation, including compression [34]. In practical words, a higher VIF score denotes greater image quality.

### 6.4. Normalized Laplacian Pyramid (NLPD)

Two essential components of NLPD are local contrast gain control and local luminance subtraction. It makes use of both a local normalizing factor and a Laplacian pyramid decomposition. When comparing the deformed picture to its reference, the resultant metric value is computed inside the normalized Laplacian domain, thereby quantifying the root mean squared error in a weight-normalized Laplacian domain. Practically speaking, better image quality is indicated by a lower NLPD score [35].

### 6.5. Feature Similarity (FSIM)

Two low-level characteristics are used by FSIM to assess picture quality. These characteristics stand for several facets of the visual system in humans. Phase congruency (PC) is a dimensionless property that is used to measure the importance of local structure. Secondly, contrast information is taken into consideration by the picture gradient magnitude (GM). FSIM is a flexible tool for evaluating many features of images because it may be used in both color and luminance versions. Superior picture quality is indicated by a higher FSIM metric value [36].

### 6.6. Peak Signal-to-Noise Ratio (PSNR)

A metric called PSNR is used to compare an image’s maximum achievable power to the amount of noise or distortion that is impacting it in order to determine the image’s quality. It basically quantifies a picture’s proximity to the perfect, spotless image with the best possible quality.

A picture’s PSNR is determined by comparing it to this perfect, clean image and measuring the power difference. The PSNR is frequently used to evaluate the performance of several image processing methods, including compressors, filters, and related apparatus. A higher PSNR value in this case denotes a more effective compression or filtering method for maintaining image quality. The PSNR is a useful statistic for assessing the effectiveness of image processing techniques since, in essence, a greater value corresponds to a better degree of fidelity to the original picture [37].

### 6.7. CIEDE2000

CIEDE2000 includes the weighting factors for the lightness, chroma, and hues in L*, a*, and b* perceptual space. It also includes factors for dealing with the relationship between chroma and hue [38].

### 6.8. VDP2

VDP2 [39] claims that it is more resilient to varying luminance circumstances and performs better on photographs taken in low light. This measure, which is stated as a mean opinion score, forecasts not only the quality deterioration with regard to the reference picture but also the visibility of changes between the original and reference photos for an average observer.

### 6.9. Butteraugli

To calculate the psycho-visual difference between two pictures, the Butteraugli metric [40] is used. Google invented this statistic. Butteraugli produces a score that only takes into account the portions of the degraded picture that are thought to include artifacts, disregarding variations that are not visually noticeable. This measure produces a heatmap that illustrates the differences between two photos in addition to a quality metric.

### 6.10. Weighted Average Deep Image Quality Measure (WaDIQaM)

The complete reference quality metric based on deep neural networks is called the WaDIQaM for full-reference IQAs [41]. The LIVE and TID2013 datasets are used to train the network end-to-end. RGB pictures are used as input for this metric, which has also been computed across all available pre-trained models. With a score between 0 and 100, a lower number indicates higher image quality.

### 6.11. LPIPS

LPIPS makes use of the fact that, even for distinct neural network topologies, deep network activations may be used as a perceptual similarity metric. By “calibrating” networks linearly—that is, by superimposing a linear layer on top of pre-made classification networks—this measure yields quality ratings.

### 6.12. Information Content-Weighted Structural Similarity Measure (IW-SSIM)

By adding the concept of information content-weighted pooling, the SSIM index can be expanded upon to the IW-SSIM [42].

The JPEG has used all these metrics for the image quality assessment of the compressed images using different JPEG standards [43].

## 7. JPEG AIC Framework

**Figure 4:**
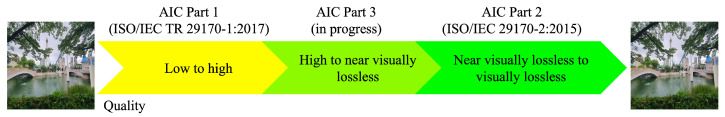
Different parts of JPEG AIC (https://jpeg.org/aic/aic3.html) (accessed on 30 November 2023).

The primary goal of the AIC initiative is to identify and assess fresh advancements in image coding research, focusing on areas such as new compression methods and quality assessment procedures. It has three parts, as shown in Figure 4.

The first two parts are the technical report, “Guidelines for image coding system evaluation” in ISO/IEC TR 29170-1:2017, and a standard titled “Evaluation procedure for nearly lossless coding” in ISO/IEC 29170-2:2015. These documents encapsulate the most effective practices sanctioned and advised by the JPEG committee. They incorporate both objective scoring and subjective evaluations to ensure that a codec’s quality assessment has undergone rigorous testing to meet the demands of global deployment.

Recently, a renewed focus on the assessment of image coding (AIC) has commenced, carrying forward the earlier standardization endeavors with the objective of crafting a new standard, termed AIC Part 3 (or AIC-3). Significantly, this initiative has identified a gap in visual quality assessments not adequately addressed by previous methodologies, particularly in the range from high to nearly visually lossless. The AIC-3 standard aims to introduce innovative criteria for evaluating images falling within this identified gap, encompassing both subjective and objective assessment techniques.

## 8. Discussion

Through a comprehensive analysis of the JPEG working groups’ review and experimental studies [43], specific metrics have emerged, showcasing a high correlation with the mean opinion scores (MOSs) given by observers. Notably, MS-SSIM, VIF, and NLPD stand out as key metrics in this regard. When focusing solely on classical compression metrics, the top-performing scores, in sequence, are Butteraugli, MS-SSIM, and VDP2.

However, in the context of AI-based compressed images, a distinct set of metrics proves to be more effective. MS-SSIM, VMAP, and VIF(P) demonstrate superior performance in this scenario. Nonetheless, it is important to note that VMAF shows a lower performance in terms of the Spearman correlation in these cases.

Overall, the dominance of MS-SSIM as a predictor of image quality across diverse compression artifacts is evident. Its consistent performance sets it apart as a clear front-runner in effectively forecasting image quality. Further information and detailed experimental results yielding these outcomes can be found in [43].

## 9. DL- and LLM-Based IQAs

As LLMs have been used in all the fields, in this section, we explored several DL- and LLM-based IQA methods classified in different categories.

### 9.1. Unified IQA

In the study [44], the researchers tackled the limitations of traditional image quality assessment (IQA) methods that typically require extensive fine-tuning for adaptation to new scenarios. They introduced a novel approach called prompt-based IQA (PromptIQA), which utilizes a small set of image–score pairs (ISPs) as prompts. This method allows the system to adapt directly to diverse assessment requirements without the need for retraining. Trained on a mixed dataset with innovative data augmentation strategies, PromptIQA demonstrated enhanced adaptability and outperformed state-of-the-art methods in terms of performance and generalization across different applications. This advancement significantly reduces the reliance on large, specialized datasets and speeds up the readiness of IQA models for practical use.

Similarly, the Q-Align study [45] presented another significant advance in the field of IQA. As the internet continued to swell with visual content, there emerged a critical need for machines capable of evaluating this content both robustly and in a manner aligned with human judgments. This study leveraged the capabilities of LMMs, which were previously shown to be effective in various related fields. The researchers adopted a novel approach by teaching these models using text-defined rating levels, simulating the subjective processes employed by human raters, who typically assess visual content based on discrete, text-defined levels rather than numerical scores.

This method, named Q-Align, achieved state-of-the-art performance in IQA as well as in image esthetic assessment (IAA) and video quality assessment (VQA). The researchers made their code and pre-trained weights publicly available, encouraging further exploration and application in the evolving field of visual content evaluation.

### 9.2. Explainable IQA

Multimodal large language models (MLLMs) have made significant progress in visual understanding, yet their potential in IQAs is still largely untapped. The paper [46] explored various prompting systems combining standardized psychophysical tests and NLP strategies to enhance MLLMs’ performance in IQAs. The authors assessed three open-source and one close-source MLLM on several visual attributes of image quality. The experimental results revealed that only the proprietary GPT-4V model somewhat approximated human perception of image quality, although it struggled with fine-grained distinctions and comparing multiple images, tasks easily handled by humans.

The paper [47] introduced VisualCritic, the first LMM designed for broad-spectrum image subjective quality assessment and capable of operating across various datasets without specific adaptations. VisualCritic demonstrated its effectiveness through extensive testing, outperforming other LMMs and traditional models in assessing and describing the quality of both AI-generated and photographic images.

The study in [48] introduced “Co-Instruct”, an open-source, open-ended visual quality comparer, trained on the new Co-Instruct-562K dataset derived from LLM-based image descriptions and GPT-4V responses. Additionally, a new benchmark called MICBench was developed for multi-image comparison among LMMs. Co-Instruct demonstrated 30% higher accuracy than leading open-source LMMs and also outperformed its “teacher”, GPT-4V, in various benchmarks.

MLLMs are rapidly evolving, yet their capability in image esthetics perception remains unclear but is crucial for real-world applications. To bridge this gap, the authors in [49] introduced AesBench, a new benchmark designed to evaluate MLLMs’ esthetic perception capacities. AesBench included an Expert-labeled Aesthetics Perception Database (EAPD) with diverse images and expert annotations and a set of criteria assessing MLLMs across four dimensions: perception, empathy, assessment, and interpretation, revealing that MLLMs currently possess only basic esthetic perception abilities.

The authors in [50] proposed DepictIQA. DepictQA descriptively and comparatively interprets image content and distortions, aligning more closely with human reasoning. The development of DepictQA involved establishing a hierarchical task framework and collecting a multi-modal IQA dataset, showing enhanced performance over traditional methods and demonstrating the potential for language-based IQA methods to be customized to individual preferences.

Multi-modality foundation models like GPT-4V have introduced a new paradigm in low-level visual perception and understanding, capable of responding to a broad range of natural human instructions. Despite their potential, these models’ abilities in low-level visual tasks remain preliminary and require enhancement. To improve these models, a large-scale subjective experiment was conducted in [51], collecting 58K detailed human feedbacks on 18,973 images, forming the Q-Pathway dataset, and converting these into 200 K diverse-format instruction–response pairs with the aid of GPT. Experimental results show that the newly created Q-Instruct enhances low-level perception and understanding across various foundational models, setting the stage for models that can evaluate visual quality like humans.

### 9.3. No Reference (NR)

NR-IQA methods are developed to measure image quality in line with human perception without a high-quality reference image. The new QualiCLIP method, a CLIP-based self-supervised approach, was proposed in [52] to overcome the limitations of relying on annotated mean opinion scores (MOSs), which hampers scalability and applicability. QualiCLIP, which aligns image–text representations to correlate with inherent image quality and does not require MOSs, achieved superior performance across several datasets and demonstrated robustness and improved explainability over supervised methods, especially in diverse real-world scenarios.

The study in [53] explored the application of large-scale pretrained foundation models to IQAs, questioning whether high-level task scaling laws apply to the predominantly low-level IQA tasks. By integrating local distortion features into a pretrained vision transformer (ViT) using a convolutional neural network (CNN) for local structure capture and training only the local distortion extractor and injector, this approach leveraged foundational model knowledge for enhanced IQA performance, demonstrating that IQA benefits from high-level features and achieving state-of-the-art results on leading IQA datasets.

In [54], the authors introduced a self-supervised approach called ARNIQA, which models the image distortion manifold to intrinsically obtain quality representations. The method involves synthetically degrading images through an image degradation model that applies sequences of distortions, training the model to maximize similarity between patches of similarly distorted images regardless of content differences, and finally, mapping image representations to quality scores using a simple linear regressor without fine-tuning the encoder. The experiments demonstrated that ARNIQA achieves state-of-the-art performance, showing improved data efficiency, generalization capabilities, and robustness over other methods.

The authors in [55] proposed CFANet, which applies a top-down methodology, allowing high-level semantic information to guide the focus towards semantically important local distortion areas. This approach included a cross-scale attention mechanism that enhances attention on key semantic regions, improving IQA performance. Tested with a ResNet50 backbone, CFANet proved to be both more efficient and competitive on various full-reference (FR)- and NR-IQA benchmarks compared to leading ViT-based methods.

In study [56], the authors introduced two novel quality-relevant auxiliary tasks designed to facilitate TTA for blind IQA: a group contrastive loss at the batch level and a relative rank loss at the sample level. These tasks were tailored to make the model quality-aware and adapt it effectively to target data. The experiments demonstrated that updating the batch normalization statistics of the source model with just a small batch of images from the test distribution could significantly improve performance.

### 9.4. Full Reference (FR)

Existing perceptual similarity metrics, which assume well-aligned images, are often sensitive to small alignment errors that are imperceptible to human eyes. The authors in [57] investigated the impact of slight misalignments on these metrics and developed a shift-tolerant similarity metric by building upon the LPIPS framework, a popular learned perceptual similarity metric. The research involved exploring various neural network elements like anti-aliasing filtering and skip connections, resulting in a new deep neural network-based metric that is tolerant to imperceptible shifts and aligns with human similarity judgments.

The authors in [58] proposed to enrich the training process by introducing comparisons between images of differing content and using listwise comparisons to give a more comprehensive perspective. Additionally, incorporating differentiable regularizers based on correlation coefficients allowed models to better adjust their quality scores in relation to each other. The effectiveness of this approach was demonstrated through evaluations on multiple benchmarks, showcasing improved training for IQA models across various distortions and content types.

The study in [59] explored SR image quality assessment (SR IQA) within a two-dimensional space that contrasts deterministic fidelity (DF) with statistical fidelity (SF), providing insights into the strengths and weaknesses of various SR techniques. Notably, traditional SR algorithms typically focus on DF at the expense of SF, whereas recent generative adversarial network (GAN)-based methods excel in SF but may underperform in DF. To address these disparities, the authors introduced an uncertainty weighting scheme that evaluated content-dependent sharpness and texture, merging DF and SF into a new quality index, the Super Resolution Image Fidelity (SRIF) index. This index showed superior performance over existing IQA models in evaluations with subject-rated datasets.

## 10. Open Research Challenges and Future Directions

Although there has been significant work on the evaluation of the image compression standards using subjective and objective methods, there are still several challenges that need to be addressed.

### 10.1. Generalized Standard Subjective and Objective Method

From the literature analysis, we observe that a specific subjective and objective method works well for one compression standard but its performance deteriorates for other standards. So, the JPEG working group is trying to develop a standardized single evaluation method that can be used for all the standards for nearly lossless purposes. In this regard, the upcoming JPEG AIC Part 3 standard will be beneficial [60].

### 10.2. No Standardized Objective Metric

Through the analysis of the literature of the studies [1,28,61,62], it is evident that the JPEG community is trying to use several quality metrics such as PSNR, MS-SSIM, IWSSIM, NLPD, CIEDE2000, FSIM, VIF, etc. In the past, PSNR and MS-SSIM were considered suitable metrics, but in recent research, other metrics such as FSIM have also performed well, but the JPEG working group is still trying to standardize optimal metrics.

### 10.3. Effect of Content Variations

Although subjective and objective methods have been used for the evaluation of compressed natural images, there is still a need for the evaluation of the methods for other images, such as remote sensing images, infrared images, medical images, etc., to observe the effect of the content on the performance of the methods.

### 10.4. Low-Power Device Compatibility

From the literature, it has been observed that most of the methods are being tested on computers or GPUs, but none of them are being evaluated on low-power devices such as tablets and mobiles, so it will be an interesting research topic for future researchers to explore the performance of the methods using low-power devices.

## 11. Conclusions

Image compression introduces multiple distortions, and these distortions impact the visual quality of the image. In this review, we summarized and discussed the impact of several JPEG image compression standards on the visual quality of the image. JPEG 1 and JPEG XL introduce blocking and softening artifacts, respectively, whereas JPEG 2000 introduces ringing artifacts. In contrast to these conventional standards, a recently developed learning-driven JPEG AI solves these problems; however, it suffers from striped region distortion at an extremely low bit rate. In this review, we discussed different subjective and objective IQA methods used by JPEG experts to find the optimal values of the IQMs for the nearly visually lossless images, which is the primary objective of the upcoming standard JPEG AIC-3. We found that MS-SSIM is still considered to be the optimal objective metric used by JPEG experts. We also found that the double-stimulus method works better for the IQA, whereas for the nearly visually lossy and lossless compression, the JPEG working group has defined a particular framework. All these are for the IQA of the specific standard. However, from the detailed analysis, it is evident that there is no general framework that can incorporate all types of distortions and artifacts introduced by the different JPEG standards. Moreover, there is still a need to explore the impact of the variation in the content in the image on the performance of these metrics. One of the interesting areas of research in this domain can be to test the methods on low-power devices such as cellular phones and tablets, as JPEG images are widely used in web browsers and on low-power devices as well. The impact of distortion on high-resolution devices can be easily detectable; however, there is an open research topic to find optimal metrics for the nearly visually lossless image compression in the context of low-power devices.

## Figures and Tables

**Table 1:** Summary of image quality assessment studies.

Study	Focus	Key Findings	Limitations
[10]	Medical Images	Comparative study of subjective methodologies.	Limited to the medical images domain.
[11]	2D and 3D Images	Comprehensive analysis of subjective and objective methods.	May not cover all possible image types and scenarios.
[12]	Images and Videos	Review of existing subjective and objective evaluation methods.	Applicability to specific image domains not discussed. Temporal limitations in findings.
[13]	Controlled Lab Setting	Comparison of three subjective quality evaluation approaches.	Limited to controlled laboratory settings.
[14]	Video Quality Ratings	Examination of six subjective video quality rating approaches.	Limited to video content assessment. Potential subjectivity in viewer preferences.
[15]	Perceptual Quality	Assessment of perceptual visual quality parameters.	May not cover all aspects of image quality perception. Subjective nature of perceptual assessments.
[17]	Visual Quality Metrics	Evaluation of visual quality metrics for perceptual coding.	Applicability to perceptual coding scenarios.
[18]	No-Reference IQA	Overview of approaches for evaluating no-reference image quality.	Limited to no-reference image quality assessment.
[19]	No-Reference IQA	Overview of methods for evaluating no-reference image quality.	Limited to no-reference image quality assessment.

## Data Availability

No new data were created or analyzed in this study. Data sharing is not applicable to this article.
